# Comprehensive Multidisciplinary Management of Bilateral Cleft Lip and Palate

**DOI:** 10.7759/cureus.52643

**Published:** 2024-01-20

**Authors:** Japneet K Kaiser, Ranjit H Kamble, Karthika Nambiar, Sumukh Nerurkar, Dhwani Suchak

**Affiliations:** 1 Orthodontics and Dentofacial Orthopedics, Sharad Pawar Dental College and Hospital, Datta Meghe Institute of Higher Education and Research, Wardha, IND

**Keywords:** multidisciplinary management, marryland bridge, abg, permanent dentition, bilateral cleft lip and palate

## Abstract

Cleft lip and palate (CLP) represent a multifaceted congenital deformity encompassing skeletal, dental, and functional discrepancies. This case report presents the management of a 13-year-old female with bilateral CLP, focusing on the challenges associated with permanent dentition and retained deciduous teeth. The patient's history included prior lip and palate repair surgeries, leading to poor aesthetics and functional concerns. A multidisciplinary approach involving orthodontics, oral surgery, and prosthodontics was implemented. Clinical examinations revealed dental abnormalities, oro-nasal fistula, and skeletal discrepancies, necessitating a comprehensive treatment plan. The orthodontic intervention aimed at aligning the dentition, followed by surgical closure of the oro-nasal fistula and alveolar bone grafting (ABG) to facilitate permanent canine eruption. Prosthetic replacement of missing maxillary lateral incisors was accomplished, enhancing aesthetics with minimal invasiveness.

Results demonstrated significant improvements in profile, dental alignment, and functional stability. Cephalometric and dental parameter analyses confirmed the corrections and enhancements achieved, affirming the success of the multidisciplinary treatment. This case report emphasizes the importance of a collaborative multidisciplinary approach in effectively addressing the complexities of bilateral CLP in patients with permanent dentition and retained deciduous teeth. The comprehensive treatment strategy rectified dental and skeletal issues and positively impacted the patient's overall well-being and self-confidence.

## Introduction

A congenital malformation known as cleft lip and palate (CLP) is linked to maxillary sagittal, transversal, and vertical differences [1,2]. This deformity frequently coexists with dental anomalies such as transpositions, hyperdontia, hypodontia, and skeletal differences. The most common is hypodontia, mainly when the maxillary lateral incisors are absent [3,4]. In addition, patients with CLP are always afflicted with malocclusion, oronasal fistula, and speech and hearing impairment. Anteroposterior and transverse maxilla deficiencies cause anterior and posterior crossbites, and incisor rotation is caused by muscle pull. Dental neglect often results in periodontal disease and carious teeth [5,6]. This case report intends to discuss the treatment performed in the case of a female presenting with bilateral CLP.

## Case presentation

A 13-year-old female and her parents presented to the Department of Orthodontics and Dentofacial Orthopaedics with a chief complaint of poor aesthetics. The patient had no previous dental history. Furthermore, the patient had a medical history of lip surgery repair at the age of 6 months and palate repair at the age of 2.5 years. There was no significant family history with similar complaints.

**Figure 1:**
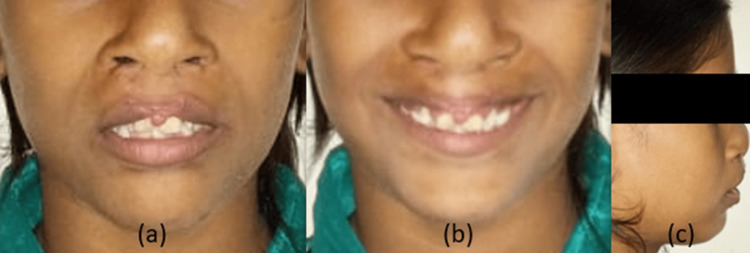
Pre-treatment extraoral photographs: (a) frontal, (b) smiling, (c) profile.

Extraoral examination showed convex profile, leptoproscopic face form and dolichocephalic head form, short upper lip, scars on upper lip from lip repair surgery, flat nasal dome, obtuse nasolabial angle, shallow mento-labial sulcus, incompetent lips. No abnormality was detected on opening or closing of the mouth, and no abnormality was detected on palpating temporomandibular joint (TMJ) (Figure 1).

**Figure 2:**
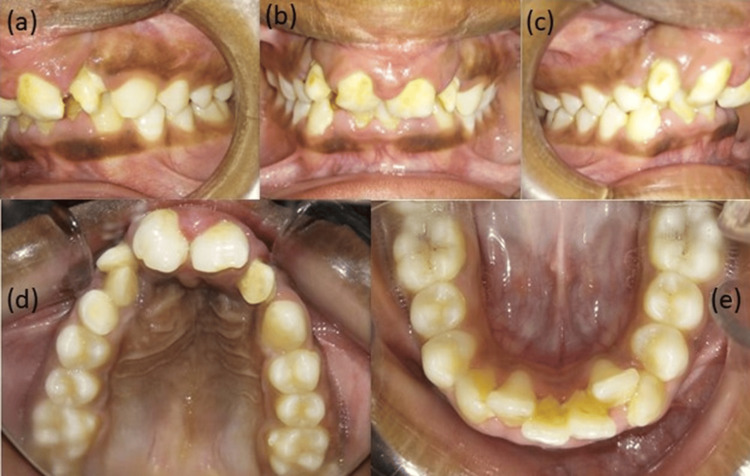
Pretreatment intraoral photographs: (a) left occlusion, (b) frontal view, (c) right occlusion, (d) maxillary arch, (e) mandibular arch.

On intra-oral examination, the upper arch had central incisors, lateral incisors bilaterally, and a retained deciduous lateral incisor and canine on the right quadrant with the absence of a permanent canine, whereas a canine was seen in the arch in the left quadrant. Both first and second premolars and first and second molars were present in both quadrants. The lower arch had all permanent dentition erupted from central incisors to second molars in both the third and fourth quadrants. Molar relation: Angle's Class I bilaterally (Figure 2).

On further examination, it was seen that both the laterals of the maxillary arch were evaluated to be peg-shaped laterals (Figure 2d) and had Grade 2 mobility. Mild crowding was present in the maxillary arch (Figure 2d), and moderate crowding was present in the mandibular arch (Figure 2e). A cleft of the palate was seen extending from the alveolus to the third rugae, involving only the hard palate and shallow vestibule (Figure 2d, 2b).

A functional examination was conducted to evaluate the oro-nasal fistula, where the patient was asked to hold water in her mouth and lean her head downwards. During this, water spilled from her nose, confirming the presence of an oro-nasal fistula that commuted water out of her nose.

**Figure 3:**
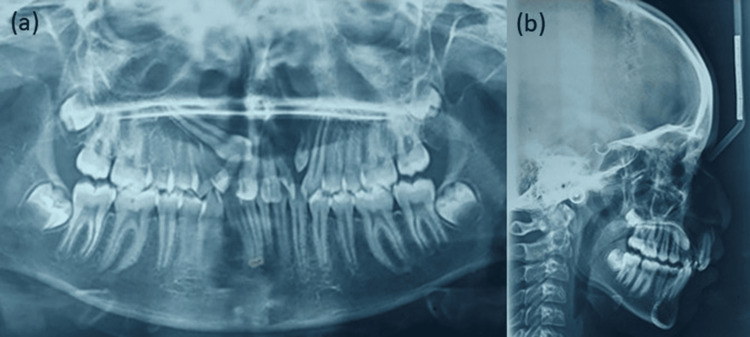
Pretreatment radiographs: (a) orthopantomogram, (b) lateral cephalogram.

Further radiographic examinations were conducted for proper diagnosis. A pretreatment orthopantomogram (OPG) revealed an impacted permanent canine on the right quadrant, incomplete root formation of peg laterals, and erupting supernumerary teeth between the peg lateral and permanent canine on the left quadrant in the maxillary arch (Figure 3a). A pretreatment Lateral cephalogram revealed a prognathic maxilla, Skeletal Class II growth pattern with an average to horizontal growth pattern, retroclined maxillary incisors, upright mandibular incisors, protruded upper and lower lips, and an obtuse nasolabial angle. Radiolucency in the maxillary arch between the premaxilla and the palate is due to the oro-nasal fistula (Figure 3b).

Cephalometric values are presented in Tables 1, 2.

**Table 1:** Cephalometric values comparing skeletal parameters of pretreatment to post-treatment lateral cephalogram. SNA: sella nasion point; SNB: sella nasion point B; ANB: point A nasion point B; Point N: point nasion; N: nasion; GO: Gonion; GN: Gnathion; SN: sella nasion; FMPA: Frankfort mandibular plane angle.

Skeletal Parameter	Normal Values	Pretreatment	Post-treatment
SNA (in degrees)	82	95	93
SNB (in degrees)	80	82	85
ANB (in degrees)	2	13	9
Point N perpendicular to point A (mm)	0+2	6	4
N perpendicular to Pogonion (mm)	0-1	-5	-3
Beta angle (in degrees)	27-35	22	24
GO-GN to SN (in degrees)	32	26	25
FMPA (in degrees)	25	25	28

**Table 2:** Cephalometric values comparing dental and soft tissue parameters with pretreatment to post-treatment lateral cephalogram. Values are calculated by the author. U1: upper central incisor; NA- nasion to point A; L1: lower central incisor; NB: nasion to point B; SN: sella nasion; E line: esthetic line.

Parameter	Normal Values	Pretreatment	Post-treatment
U1-NA (in degrees)	22	-15	10
L1-NB (in degrees)	25	20	19
U1 to SN (in degrees)	102	76	92
E line to upper lip (mm)	-2	3	1
E line to lower lip (mm)	0	4	4
Nasolabial angle (in degrees)	90-100	130	110

Treatment progress

**Figure 4:**
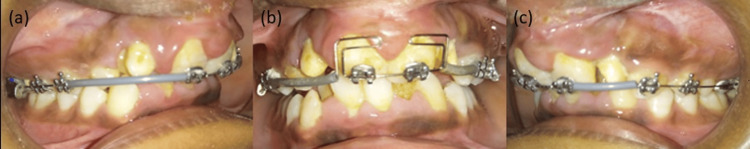
L-loop appliance in the patient's mouth for alignment: (a) right occlusion, (b) frontal view, (c) left occlusion.

The patient was advised to undergo orthodontic treatment first. Following this, 0.022" slot MBT brackets were bonded on the maxillary arch's permanent dentition only, except for the peg laterals. A 0.016 AJW arch wire was used to fabricate an L-loop that was placed bilaterally distal to the central incisor brackets; it was used to align the upper arch and procline the maxillary centrals (Figure 4). Once the upper arch was leveled and aligned, the patient was evaluated for fistula closure and alveolar bone grafting (ABG).

Before the surgical intervention, extraction of the peg laterals was performed due to their poor prognosis with supernumerary teeth, and the over-retained deciduous canine and lateral were also extracted.

**Figure 5:**
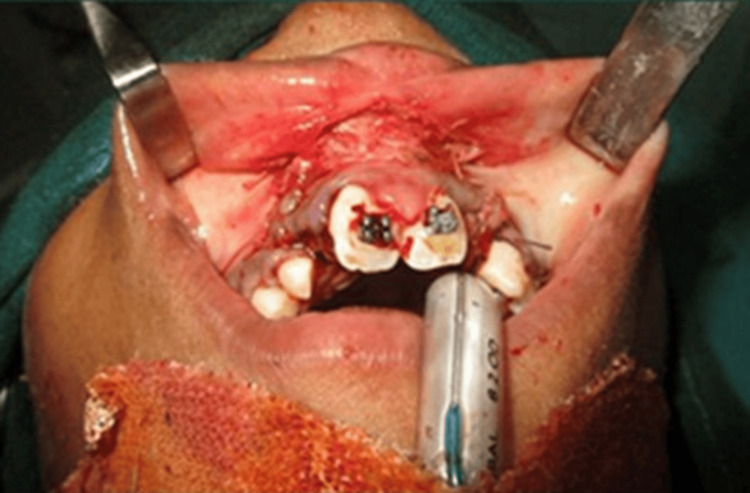
Fistula closure and vestibuloplasty.

Initially, fistula closure and vestibuloplasty were conducted simultaneously, as seen in Figure 5. This was followed by the planning of ABG within four months. ABG was planned early in the treatment as canine root formation was nearly complete, as seen on the OPG (Figure 3a). To facilitate its eruption into the arch, bone grafting was required. Hence, ABG was planned by the oral surgery team at the initial stages of the treatment.

**Figure 6:**
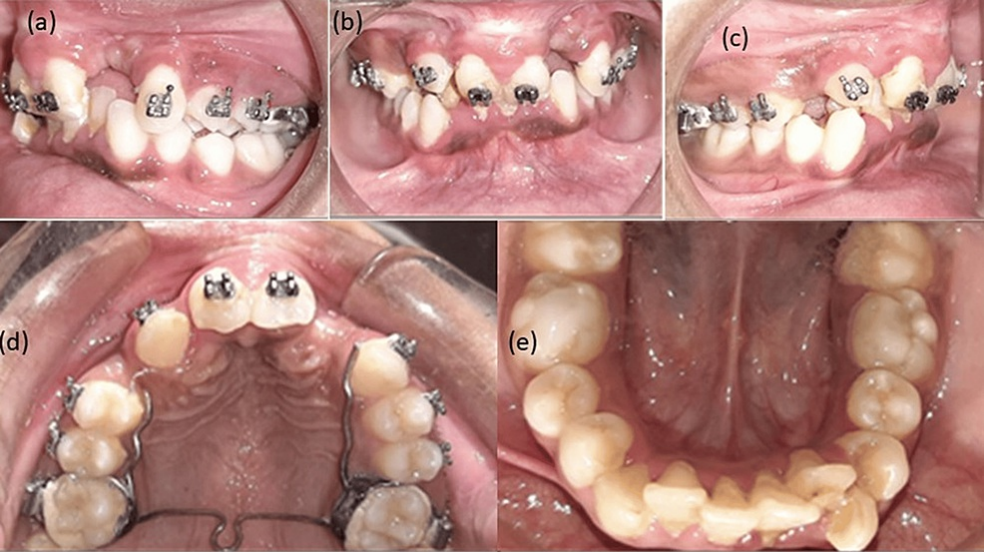
Mid-treatment photographs with a cemented extended transpalatal arch (TPA): (a) left occlusion, (b) frontal view, (c) right occlusion, (d) maxillary arch with fixed extended TPA, (e) mandibular arch. Pictures are taken by the author.

Before the ABG procedure, extended TPA was cemented in the maxillary arch as shown in Figure 6d.

**Figure 7:**
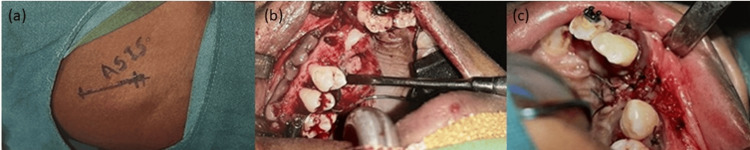
Alveolar bone grafting procedure: (a) donor site, (b) recipient site, (c) after closure.

This was done to prevent the arch from collapsing during the healing period due to scar formation. Surgical repair of the naso-alveolar fistula was performed using a cortico-cancellous iliac bone graft from the left side under general anesthesia (Figure 7).

Immediately after the ABG, the patient discontinued treatment for one year due to a spike in COVID-19 cases that year. Once the patient was comfortable visiting the hospital again, it was observed that her canine was well erupted in the arch, indicating the success of the ABG in achieving the main objective of the start of treatment and also to close the oro-nasal fistula (Figure 6).

**Figure 8:**
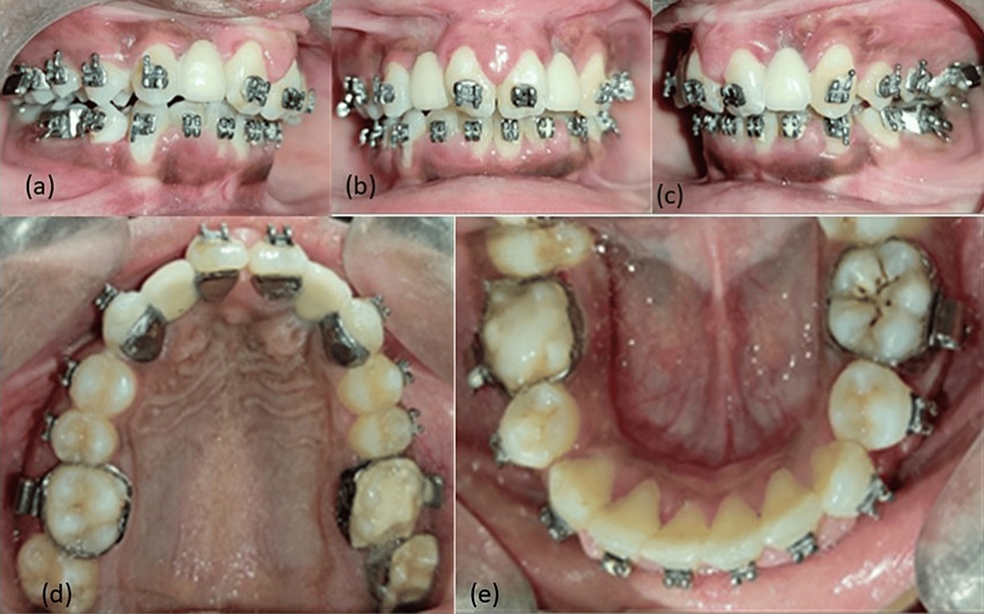
Prosthetic replacement of lateral incisors using the Marryland bridge: (a) right occlusion, (b) frontal view, (c) left occlusion, (d) maxillary arch, (e) mandibular arch.

Since approximately one year had passed since the ABG, orthodontic treatment was resumed immediately after the patient reported back to the Department of Orthodontics and Dentofacial Orthopaedics. The lower arch was also bonded this time, and extraction of both right and left mandibular first premolars was done to relieve mandibular arch crowding. The maxillary right quadrant canine was also distalized to create space for the prosthetic replacement of the extracted lateral incisors. After the alignment of both the maxillary and mandibular arches, the patient was sent to the Department of Prosthodontics for the replacement of both maxillary lateral incisors. An implant was initially planned for the replacement of the missing laterals. However, the patient was reluctant to undergo any further invasive procedures, so a resin-bonded FPD was suggested. This option also required minimal tooth preparation, and the patient was satisfied with this treatment choice. Tooth preparation was done on the lingual aspect of the maxillary canines and central incisors, followed by which impressions were recorded to fabricate a Maryland Bridge. Metal-ceramic prosthetic lateral incisors were fabricated and cemented into the arch within a period of 15 days (Figure 8).

**Figure 9:**
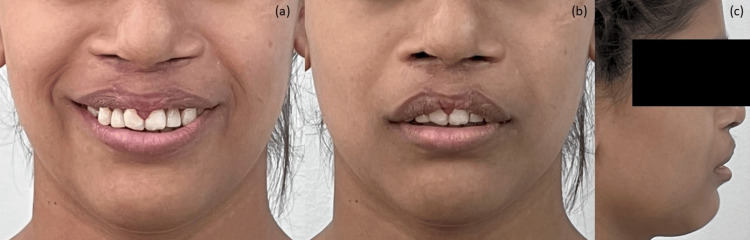
Post-treatment extraoral photographs: (a) smiling, (b) frontal, (c) profile.

**Figure 10:**
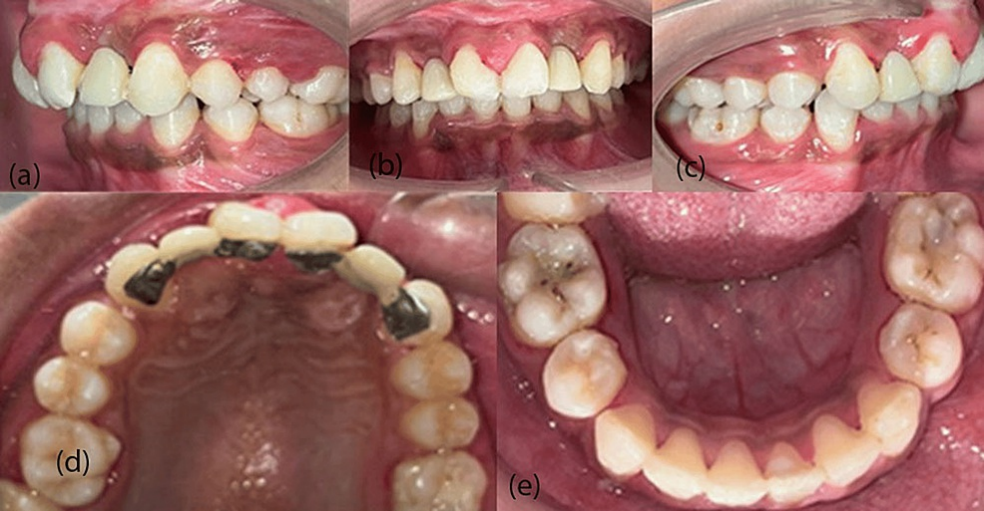
Post-treatment intraoral photographs: (a) left occlusion, (b) frontal view, (c) right occlusion, (d) maxillary arch, (e) mandibular arch.

**Figure 11:**
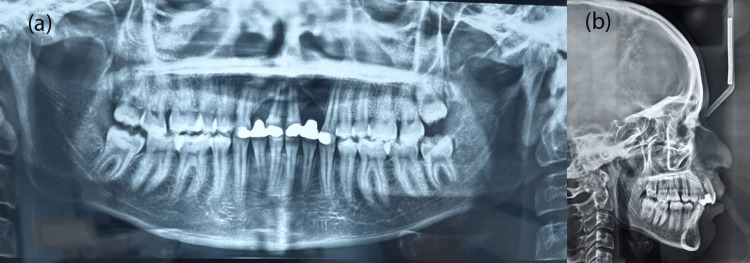
Post-treatment radiographs: (a) OPG, (b) lateral cephalogram.

Most objectives for both the maxillary and mandibular arches were achieved, and cephalometric analysis comparison also showed significant corrections. After three months of settling the occlusion, the patient was satisfied with the treatment outcome and requested the removal of the appliance. Consequently, the patient's appliance was debonded (Figures 9, 10, 11).

Results

The patient showed a significant difference in pre- and post-treatment features. Cephalometric values demonstrated substantial correction in comparison to the pre- and post-treatment analysis (Tables 1 and 2).

Profile correction was observed, with a change from a convex to a much straighter profile. The patient's smile improved because of the vestibuloplasty. The ABG reduced the severity of the maxillary prognathism. The successful surgical correction and ABG aided in the eruption of the permanent canine. Intraorally, well-aligned arches were observed. A functionally stable occlusion was achieved, and the Class I molar relation was retained. The midline was corrected.

Prosthetic replacement with a Maryland bridge enhanced the patient's aesthetics with the least surgical intervention, as requested by the patient. The patient was very satisfied with the results. She showed improvement not only clinically but also psychologically.

## Discussion

Managing bilateral CLP in patients with permanent dentition and over-retained deciduous teeth presents a complex scenario that requires a coordinated effort among orthodontists, oral surgeons, and prosthodontists. This situation demands a comprehensive treatment plan to address the challenges associated with dental alignment, occlusion, skeletal discrepancies, and prosthetic rehabilitation [7].

Orthodontists play a crucial role in managing patients with bilateral CLP who have permanent dentition and retained deciduous teeth. Their primary focus involves correcting dental misalignments, addressing malocclusion, and managing the transition from deciduous to permanent dentition. Orthodontic treatment modalities, such as braces, aligners, and space management techniques, aim to guide the eruption of permanent teeth and correct their alignment within the cleft-affected arches. Coordination between the orthodontist and oral surgeon is crucial to plan and execute any necessary surgical interventions to aid tooth eruption or the extraction of retained deciduous teeth [8].

Oral surgeons are essential in addressing skeletal and dental discrepancies in patients with bilateral CLP and over-retained deciduous teeth. Surgical interventions may include procedures to expose impacted permanent teeth, removal of over-retained deciduous teeth, or orthognathic surgery to correct jaw alignment issues. Additionally, in cases where permanent teeth are absent or severely malformed, the oral surgeon collaborates with prosthodontists to plan for dental implants or prosthetic solutions [9].

Prosthodontists play a vital role in the rehabilitation of patients with bilateral CLP and over-retained deciduous teeth. They focus on restoring missing or malformed teeth, enhancing oral function, and improving aesthetics. Prosthodontic interventions may involve designing and fabricating dental prostheses, such as bridges, dentures, or implants, to replace missing or malformed permanent teeth. These prosthetic solutions aim not only to restore oral function but also to improve the patient's overall appearance and self-confidence [10].

The collaborative effort among orthodontists, oral surgeons, and prosthodontists is critical in developing a comprehensive and coordinated treatment plan for patients with bilateral CLP and retained deciduous teeth. Timely communication and synchronized treatment sequencing among these specialists are essential to address the multifaceted challenges posed by the condition [7].

Moreover, patient education and psychological support play a significant role in managing these cases. Patients and their families should be well informed about the treatment plan, expected outcomes, and the importance of compliance with orthodontic and surgical interventions. Additionally, psychological support is essential to address any emotional concerns and ensure that the patient feels supported throughout the treatment process [8].

## Conclusions

To conclude, managing bilateral CLP in patients with permanent dentition and retained deciduous teeth necessitates a cohesive partnership among orthodontists, oral surgeons, and prosthodontists. Their collective expertise ensures comprehensive treatment, addressing dental alignment, skeletal issues, and prosthetic rehabilitation. This collaborative approach aims to optimize oral function, aesthetics, and overall well-being, providing patients with improved quality of life and renewed self-confidence.
